# KRAS RENAISSANCE(S) in Tumor Infiltrating B Cells in Pancreatic Cancer

**DOI:** 10.3389/fonc.2018.00384

**Published:** 2018-09-19

**Authors:** Qingda Meng, Davide Valentini, Martin Rao, Markus Maeurer

**Affiliations:** ^1^Division of Therapeutic Immunology (TIM), Department of Laboratory Medicine (LABMED), Karolinska Institutet, Stockholm, Sweden; ^2^Centre for Allogeneic Stem Cell Transplantation (CAST), Karolinska University Hospital, Stockholm, Sweden

**Keywords:** pancreatic cancer, KRAS mutations, antibody reactivity, tumor-infiltrating lymphocytes, immunotherapy, B cells

## Abstract

KRAS is a driver mutation for malignant transformation. It is found in 30% of all cancers and in 90% of pancreatic cancers. The identification of small molecules selectively inhibiting KRAS mutants has been challenging, yet mutant KRAS has recently been shown to be targeted by tumor-infiltrating lymphocyte (TIL)-derived T cells that confer tumor regression upon adoptive transfer. Furthermore, a human IgG1 monoclonal antibody interfering with mutant KRAS function inside the cell has been described to inhibit growth of KRAS-mutant xenografts in tumor-bearing mice. B cells have been described to infiltrate pancreatic cancer and may be associated with tertiary lymphoid structures associated with good prognosis, or, in contrast, promote tumor growth. However, their function, nor their antigen-specificity has been clearly defined. We discuss here the presence of tumor-infiltrating B cells (TIB) in patients with pancreatic cancer that produce KRAS-mutant specific IgG, underlining that intratumoral T and B cells may exclusively target mutant KRAS. KRAS-specific IgG may, therefore, serve as a readout of the activation of both arms of the anti-tumor adaptive immune armament although some B-cell populations may promote tumor progression.

## Background

The Kirsten Rat Sarcoma 2 Viral Oncogene Homolog (KRAS) gene and its product have had their renaissance several times. The most recent highlight came from the unsuspected identification of the association of mutant KRAS (in endothelial cells) driving arteriovenous malformation (AVM) in the CNS (1). The first KRAS renaissance, after the original description of mutant KRAS forms as a protooncogene (2), is manifested in the observation that mutant KRAS is found in approximately 30% of all human cancers. Lung (3) and gastrointestinal cancers commonly harbor KRAS mutations, with 90–95% of pancreatic cancer lesions (particularly pancreatic ductal adenocarcinoma (PDAC)) carrying a point mutation at the G12 locus (4), the most common being KRAS_G12D_ and KRAS_G12V_. With the advent of novel therapies targeting the epidermal growth factor receptor (EGFR), the focus once again shifted to KRAS as a predictor of response to cancer therapy. Patients with KRAS-wild type colorectal cancer (CRC) will most likely, with a few reported exceptions, become refractory to targeted therapy (5). Nakadate and colleague reported that CRC cells harboring a KRAS-G13 mutation display resistance to cetuximab (anti-EGFR IgG1)-mediated antibody-dependent cellular cytotoxicity (ADCC) which involves Fas-FasL interaction (6). The heterogeneity and evolution of intra-tumoral KRAS mutations remains to be addressed in greater depth. Detailed analysis of tumor heterogeneity, measured as the tumor mutational burden (TMB), showed that up to 69% of all somatic mutations are not detectable across different tumor regions (7). Not surprisingly, tumor cell subpopulations with diverse KRAS mutations exist either within the same tumor lesion, or at different anatomical sites [for a review see Ref (8)]. KRAS mutations undermine EGFR-targeted therapies (5, 9) and variant KRAS-mutant tumor cells contribute to the outgrowth of EGFR-resistant tumor cell populations. Yet there is a different aspect. KRAS has been considered a viable tumor vaccine target, since mutant KRAS is exclusively present in cancer cells except for rare, dispersed cells within healthy colon tissue, although the direct effect of mutant KRAS in the oncogenesis of human and murine pancreatic cancer was recently addressed using CRISPR/Cas9-genome editing (10). In this study, Muzumdar et al. showed that KRAS deficiency in pancreatic cancer cells, achieved by inactivation of the gene by means of CRISPR/Cas9 excision, reduced cell growth kinetics in some PDAC cells lines and increased their dependence on phosphoinositide-3-kinase (PI3K) and downstream the mitogen-activated protein kinase (MAPK) activity for proliferation. As such, KRAS absence was also shown to sensitize PDAC cells to pharmacological PI3K/mammalian target of rapamycin (mTOR) inhibition.

To date, anti-KRAS vaccine studies have yet to deliver clinically promising results. However, a pivotal report from the Steven Rosenberg group has offered strong evidence for KRAS-directed immunotherapy: a patient with CRC exhibited clinically relevant remission upon infusion of tumor-infiltrating lymphocytes (TIL) targeting a mutant KRAS epitope (harboring the G12D mutation) restricted by HLA-C^*^w0802 (11). Furthermore, transgenic expression of the nominal T-cell receptor (TCR) targeting the KRAS mutant in recipient immune effector cells resulted in observable tumor regression: anti-KRAS_G12D_ T-cell responses cleared all mutation-positive metastatic lesions expect for one which lost the HLA-C^*^08:02 gene. Thus, mutant KRAS-specific T cells residing within tumor lesions can elicit clinically beneficial effects if the matching mutant KRAS epitope is naturally processed and presented (11). The patient's own immune cells have, therefore, been shown to target mutant KRAS, which was previously considered a “non-druggable” target. In addition to T cells, B cells can also target transformed cells expressing tumor-associated antigens (TAA) i.e. NY-ESO-1. This TAA was identified using serological analysis of recombinant cDNA expression libraries (SEREX), where antibodies are used to screen a protein expression library for specific reactivity (12–14). *In vitro*-cultivated, tumor-infiltrating B cells (TIBs) from non-small cell lung cancer (NSCLC)—which commonly harbors KRAS and EGFR mutations (3)–are able to present antigen to and activate CD4 TIL, while some TIB subsets can have an equally immunosuppressive effect on CD4+ T cells (15). Strong B-cell responses are often associated with strong T-cell responses, as in the context of anti-NY-ESO-1 reactivity (16–18) and as clinically observed in gastric cancer (19, 20). As such, the role of B cells in mediating anti-tumor immune responses due to their ability to produce cytokines as well as differentiate into antibody-secreting plasma cells in addition to professional antigen-presenting capacity has more recently received increased attention, even as a viable candidate for immunotherapy (21–24).

Unlike NY-ESO-1, efforts have been disappointing in identifying serum antibodies against KRAS in patients with a KRAS-positive malignancy. This may be possibly due to lower amounts of mutant KRAS protein expressed and subsequent lower antigen turnover in the tumor as compared to NY-ESO-1 expression, which is quite high based on immunohistological studies (25). Nevertheless, a proof-of-concept study in mice harboring various KRAS-mutant tumor xenografts showed that an anti-mutant KRAS antibody (RT11) in able to penetrate tumor cells and inhibit KRAS-mutated tumor-cell proliferation and disease progression (26).

## The PDAC tumor microenvironment (TME)

Cancer cells proliferate and sustain growth in a complex network of non-transformed cells that may inhibit or promote cancer progression. In PDAC, this phenomenon engages a multilayer interaction between the tumor cells, stromal compartment as well as immune cells which infiltrate the tumor (27, 28). Antigen-specific CD4 and CD8 lymphocyte infiltration into pancreatic tumor lesions are generally viewed to be a positive prognostic factor for survival (29). In contrast, the accumulation of CD4+ CD25^hi^ regulatory T cells (Tregs) contribute to immunosuppression in the TME and blunt productive anti-tumor responses, as demonstrated by depletion studies in preclinical models of PDAC (30–32). Transforming growth factor beta (TGF-β), a crucial biological mediator associated with Tregs, promotes fibrosis in the TME, which in turn inhibits the cytolytic function of cytotoxic CD8 T cells in addition to promoting tumor growth (33). There is also evidence that PD-L1-expressing TCR gamma delta (γδ) T cells can abrogate the tumor-directed activity of CD4 and CD8 T cells, which can be reversed by immune checkpoint blockade using anti-PD-L1 antibodies (34). Among non-immune cells are fibroblasts and different types of epithelial cells that contribute to the tumor architecture, maintain close reciprocation with immune and tumor cells and are associated with prognosis of patients with cancer (35).

An important characteristic of chronic immune activation in chronic infections as well as cancer is the development of tertiary lymphoid structures or organs (TLS or TLOs, respectively) containing B and T cells. This has been identified also in patients with pancreatic cancer; intra-tumoral rather than peri-tumoral TLOs with cellular infiltrates encompassing B and T cell populations in conjunction with lower immunosuppressive Tregs and increased expression of IFN-γ, IL-12, TBX21 (Th1-specific transcription factor) and IL-23 were associated with improved disease-free survival (*p* = 0.016) (36). However, CD1d^hi^ CD5+ B cells which produce IL-35, in fact, contribute to the pathogenesis of pancreatic cancer and tumor progression, as shown in a mouse model of PDAC (37). Although PDAC is thought to exhibit an immunosuppressive microenvironment, the presence of cytotoxic immune cells proximal to transformed cells has been reported to be associated with increased patient survival despite desmoplastic histology in the tumor texture (38). T cells directed against pancreatic cancer cells by recognizing the patient's “private mutations” (private neoantigens) (39, 40) or against cancer-associated antigens, e.g., mesothelin (41, 42) and survivin (43), have been reported by various laboratories and demonstrate the possibility to successfully isolate and expand tumor-reactive T cells from pancreatic cancer specimens (39, 43, 44). Collectively, these findings suggest that although present in the TME, tumor-reactive T cells are unable to contain tumor progression due to unfavorable conditions therein.

## Antibodies against KRAS in pancreatic cancer-associated B cells

Anti-tumor cellular immune responses may be directed against: (i) non-mutant but overexpressed targets i.e. mesothelin, NY-ESO-1, survivin, (ii) private mutated proteins (neoantigens) as well as (iii) mutant targets that are shared among different individuals (shared neoantigen), such as KRAS (39). T cells infiltrating into PDAC and can recognize TAAs tend to be positively associated with patient survival—this observation is likely applicable to T cells targeting private mutations also (39, 45). However, the question of whether anti-PDAC antibodies are present—either circulating (serum-bound) or within the TME—remains to be fully addressed. Of note, *bona-fide* mutant KRAS-specific T cells have been primarily identified *in situ*, i.e., in the tumor lesion, and *not* in the peripheral circulation. This notion can now be complemented with anti-KRAS antibodies *in situ*.

**Figure 1:**
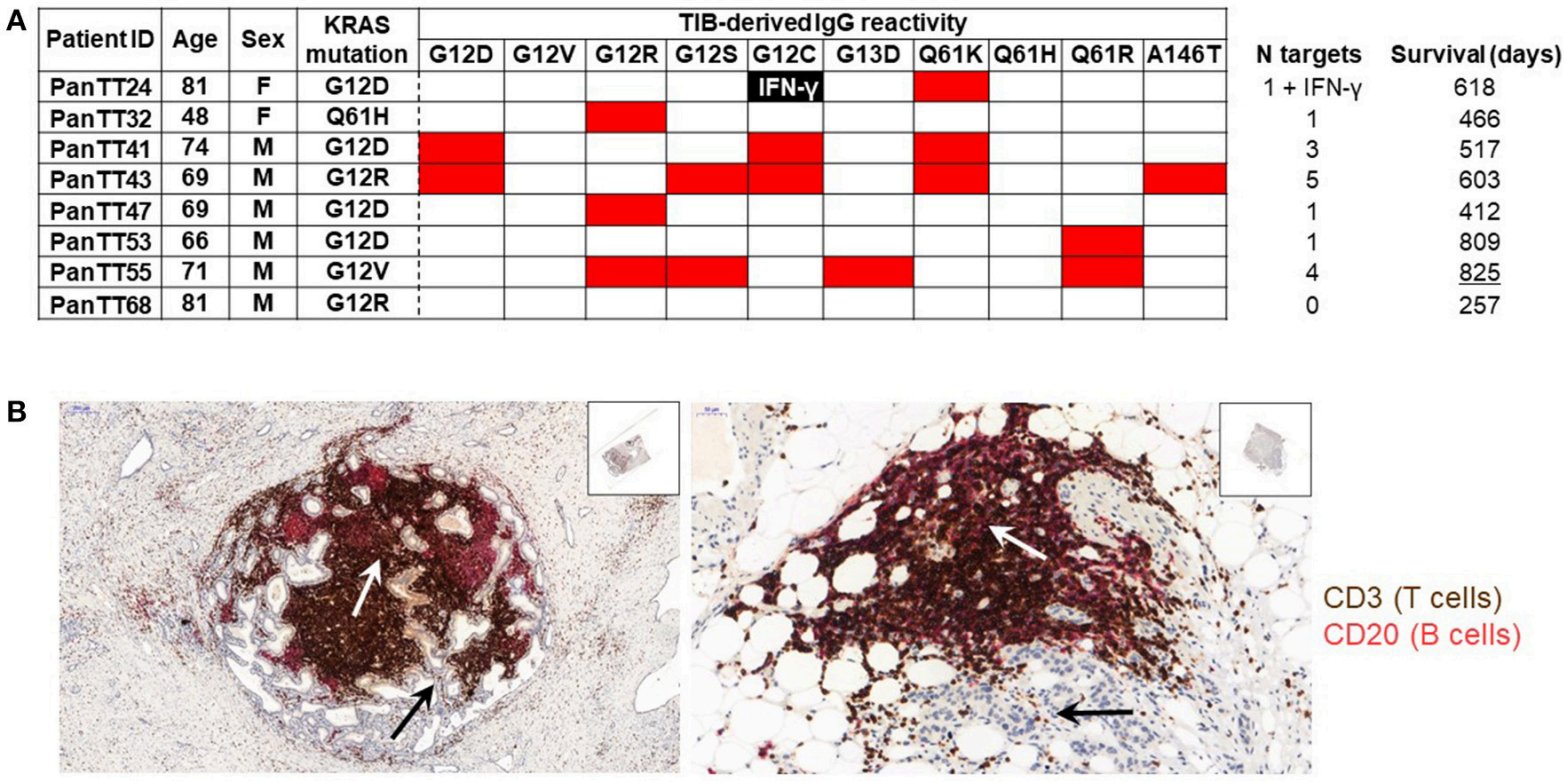
Recognition of mutated KRAS targets by patients' TIB-derived IgG. **(A)** TIB supernatants from eight patients with pancreatic adenocarcinoma (PDAC) were tested for IgG reactivity to KRAS mutated peptides (and the corresponding wildtype sequences) on a peptide microarray chip. Shown is a heat map, wherein red indicates a positive response while the blank boxes indicate the absence of a response. TIB-IgG reactivity to mutated KRAS targets appeared to be generally diverse. None of the TIB supernatants showed reactivity to any of the wildtype KRAS sequences, while only TIB-IgG from patient PanTT41 recognized the endogenous KRAS mutation. **(B)** Immunohistochemistry photos from representative pancreatic cancer tissue sections, focusing on the center of the tumor mass. The white arrows point to the tumor parenchyma (thus, the tumor cells within the PDAC lesion), while the black arrows point to clustering of T (brown) and B (pink/red) cells at the same location within the tumor parenchyma, indicating that lymphocytes are in close physical contact with the cancer cells. Details of the immunohistochemical staining protocol are included in the “Materials and Methods” section of the Supplementary Material. The Table indicates the patients ID (right column), gender and age, as well as the summary of the number of mutant KRAS targets recognized / TIB IgG sample and the survival time (after primary diagnosis). IFNγ production directed against a mutant KRAS epitope was observed in a single patient. Isolation and *in vitro* expansion of pancreatic cancer TIL in the presence of IL-2, IL-15, and IL-21 was performed as previously described (43, 56). TIB cultures were generated by culturing each tumor tissue fragment in 24-well plates containing 1 ml TIB medium (70% Cellgro GMP-grade serum-free medium (CellGenix), 20% B95-8 supernatant containing EBV virus (filtered with 0.22um filter), 10% FBS, penicillin (100 IU/mL), streptomycin (100 mg/mL; Life Technologies) and amphotericin B (2.5 mg/L; Sigma-Aldrich). Once a stable TIB cell line was established, the cultures were replenished with and maintained in TIB medium without the B95-8 supernatant.

TIL encompass not only T cells, but also B cells: their contribution to anti-tumor immune responses, however, was described to be negative. More recent studies show that some TIB subsets appear to be involved in promoting pancreatic tumorigenesis and cancer development in association with co-expression of CD1d and CD5 on their surface, IL-35 production as well as loss of intracellular expression of hypoxia-inducing factor 1 alpha (HIF-1α) (37, 46). Furthermore, TIBs expressing Bruton tyrosine kinase (BTK) can impede productive T-cell responses in the TME, although this immunosuppressive activity is pharmacologically reversible with ibrutinib (47). Thus, one might ask if there is a positive side to B cells in PDAC. We, therefore, tested the antigen specificity of PDAC-infiltrating B cells (TIB) by first isolating them from tumor tissue, immortalizing them using Epstein-Barr virus (EBV) infection and assessing their IgG production against wild type and mutant KRAS targets. Tumor tissue samples were obtained from eight patients with PDAC for generating TIL cultures comprising B and T cells separately, and the study was approved by Regional Ethics Review Board (Regionala etikprövningsnämnden) at Karolinska Institutet, Sweden (EPN: 2013/576-31 & 2013/977-31/1). In addition, the patients also provided written informed consent. TIB supernatant samples in serum-free medium (no human serum or FBS) were incubated in three 12-plex peptide microarray chips [format described in (48), which was previously reported from our group for human serum IgG reactivity to the H1N1 influenza proteome] embedded with 12-mer peptides from the human KRAS protein (UniProt ID: P01116) covering the most frequently occurring point mutations associated with cancer: G12D, G12V, G12R, G12S, G12C, G13D, Q61K, Q61H, Q61R, and A146T. The corresponding wild type/non-mutated sequences were also included. PDAC-infiltrating T cells had been expanded *in vitro* with IL-2, IL-15, and IL-21 and were later tested for KRAS wild type and mutant recognition defined by IFN-γ production. Genetic analysis, using a commercial kit, showed that 7/8 pancreatic cancer specimens (87.5%) carried a mutation at the G12 position. 4/7 patients (57.1%) had the KRAS_G12D_ mutation, two patients carried the KRAS_G12R_ mutation (25%), one patient tested positive for the KRAS_G12V_ mutation and tumor material from another patient harbored the KRAS_Q61H_ mutation. Wild type KRAS peptides were not recognized by any of the TIB-IgG samples. Only a single patient had TIB-IgG that recognized the endogenous KRAS mutation (PanTT41, KRAS_G12D_; Figure 1A). TIB-IgG from the same patient also recognized two other KRAS mutations, namely KRAS_Q61K_ and KRAS_G12C_. Although TIB-IgG from patient PanTT43 did not recognize the autologous KRAS mutation (G12R), reactivity to five other KRAS mutations was observed. TIB-IgG from patient PanTT55, positive for the KRAS_G12V_ mutation, also recognized four non-endogenous KRAS mutations (G12R, G12S, G13D, and Q61R). Only a single mutated KRAS target was recognized by TIB-IgG from 4/8 patients, while none of the KRAS targets tested were recognized by TIB-IgG from patient PanTT68. In general, mutations occurring at position G12, which cover approximately 98% of human PDAC tumors, were most frequently recognized by TIB-IgG from 5/8 patients tested. Representative photographs of immuno-histological staining from two patients with PDAC showed that T- and B-cell infiltration into the center of the pancreatic tumor tissue cluster together, indicating these cells are in close contact with the tumor parenchymal cells (Figure 1B). Only 1/3 T-cell cultures (expanded from TIL) recognized mutant KRAS_G12C_ (PanTT24) but not the patient's endogenous KRAS_G12D_ mutation after repeated exposure to the autologous tumor cell line (Meng et al. manuscript accepted for publication in the British Journal of Cancer, July 2018).

## Perspective for KRAS-directed Ig in tumor-infiltrating B cells

These data show that, akin to tumor-infiltrating T cells, B-cell responses exclusively targeting mutant and not wildtype KRAS are present in the TME yet not detectable in serum. Whether the recognition of non-endogenous (except in 1/8 specimen) KRAS mutations represents an ongoing or a past evolution of cancer cells with mutant KRAS (8) has to be evaluated. Like T-cell responses (11), immunological fine-tuning of antibody reactivity to cancer-associated mutated proteins in the TME may qualify TIBs as a viable source for the molecular blueprint of potential anti-tumor antibodies targeting neoantigens. TIBs bearing KRAS-specific BCRs could represent the cross-activation of the humoral immune response in patients with KRAS-mutant cancer the same way as the NY-ESO-1 paradigm in melanoma (12, 13, 16, 17, 25, 49, 50). The potentially tumor-promoting role of PDAC could be outsmarted, particularly if KRAS-directed B cells promote tumor progression upon binding of their BCR to the nominal target antigens and subsequent production of tumorigenic cytokines. Importantly, mutant KRAS-directed IgG responses may also indicate co-amplification of the T-cell response reflecting antigen processing and presentation by specific B cells to enhance immune surveillance even when antigen concentration may be limiting (51). The single-chain variable fragment (scFv) of specific anti-mutant KRAS antibodies may also serve as a scaffold for intracellular immunotoxins (26), which can be delivered via genes encoding antibody sequences packaged in liposomes or attached to nanoparticles (52). In a phase I/II clinical trial involving patients with locally advanced pancreatic cancer, RNA interference-based molecular therapy targeting KRAS_G12D_ as an adjunct to first-line chemotherapy was shown to be well-tolerated, with all patients displaying stable disease and two individuals even exhibiting a partial response (53). Furthermore, increased sensitivity to EGFR inhibition (54) and “druggability” of the focal adhesion kinase (FAK)—a crucial determinant of PDAC growth and aggressiveness (55)—is also achievable by targeting mutant KRAS. More cycles of KRAS renaissance are to be expected as biologically relevant tools for the targeted specific immunological therapy of patients with KRAS mutant cancer.

## Author contributions

QM performed experiments. DV was responsible for statistical analysis. MR wrote the initial draft, compiled and interpreted data, and assisted with the revision. MM initiated the work, conceptualized the information in the draft and wrote the manuscript.

### Conflict of interest statement

The authors declare that the research was conducted in the absence of any commercial or financial relationships that could be construed as a potential conflict of interest.
