# Supplementary material for: KRAS RENAISSANCE(S) in Tumor Infiltrating B Cells in Pancreatic Cancer
Source: Front Oncol. 2018 Sep 19;8:384. doi: 10.3389/fonc.2018.00384 (PMC6156365; doi:10.3389/fonc.2018.00384)
Supplement: Supplementary file 1 [file Data_Sheet_1.PDF]

SUPPLEMENTARY APPENDIX

Peptide microarray data processing.

Each KRAS peptide was represented seven times on the microarray chip. Detection of the TIB-IgG immunoreactivity to the embedded peptides was performed with an Alexa Fluor® 647 AffiniPure Goat Anti-Human Fcγ Fragment Specific IgG (Jackson ImmunoResearch). The image reading software SlideViewer (developed by Roche, in-house) was used for measuring each spot's intensity to produce recognition data matrices.

The data was preprocessed using the RMA-background correction method [1], within-array normalized using *loess* spatial correction[2] and log2 transformed. We did not implement inter-array normalization as the comparison between arrays or array groups lies beyond the scope of the present study. An average of the respective signal intensities of IgG recognition of the KRAS peptide repeats were obtained for each sample. Two matrices were then produced: (i) displaying each individual spot's intensity mean values and (ii) showing standard deviation values. We also computed the Coefficients of variation (CV); peptides with a CV of >1 were identified as large and flagged for each sample. After averaging, a panel of 2882 unique peptides was obtained for each chamber.

For each sample, the robust zeta scores ($z = (x - median(x)) / mad(x)$) were separately computed. KRAS peptides displaying very high IgG recognition were identified by setting a detection cut-off equaling 3 times the standard deviation of the zeta value of each patient sample.

Generation of tumor-infiltrating lymphocytes (TIL) from PDAC tissue

Briefly, tumor tissue samples from surgical resections were first cut into small pieces of 1-2mm^3 with a sterile scalpel. The fragmented tumor pieces were then washed twice with cold 1x PBS and placed in 24-well plates containing T-cell medium (Cellgro GMP-grade serum-free medium (CellGenix, Freiburg, Germany) with 5% pooled human AB serum (Innovative Research, Novi, MI), supplemented with recombinant human cytokines (Prospec, Ness-Ziona, Israel): IL-2 (1000IU/ml), IL-15 (10ng/ml) and rhIL-21 (10ng/ml) for culture. T-cell medium was replenished as necessary. Allogeneic PBMCs irradiated at 55Gy were used as feeder cells and added to the growing TIL at a ratio of 1:10 (feeders:TIL) after seven days of initiating the culture. The TIL were then transferred to six-well plates once more than 70% confluence was observed in the 24-well tissue culture plates, and further expanded in G-Rex flasks (Wilson Wolf, St. Paul, MN) with the addition of 30 ng/ml OKT3 (BioLegend, San Diego, CA) as well as irradiated allogeneic feeder cells at a 1:5 ratio.

Immunohistochemistry

Resected pancreatic tumor tissue specimens (from surgery) were first fixed in 4 % formalin, and then embedded in paraffin, sectioned for 4 µm tissue slices and processed for immunohistochemistry. A specialist pancreatic pathologist (Dr. C. F. Moro) selected the best quality tissue slides representing the middle of the tumor. To preserve the relative spatial distribution of the lymphocyte populations, chromophore-conjugated antibodies were combined into following. 2-plex immunostaining for preserving the spatial distribution of the T and B-cell populations were performed using the anti-human CD3 (clone LN10, Novocastra) and anti-human CD20 (clone L26, Dako) antibodies and a Leica BOND III automated immunostainer (Leica). 3,3'-diaminobenzidine (DAB - brown) was used as detection substrate for the primary antibody while alkaline phosphatase (AP - red) was used for detecting the secondary antibody.

REFERENCES

1. Irizarry RA, Hobbs B, Collin F, et al. Exploration, normalization, and summaries of high density oligonucleotide array probe level data. Biostatistics 2003;4:249-64.

2. Cleveland WS, Grosse E, Shyu WM. Local regression models. Statistical models in S 1992;2:309-76.
